# A Cloud-Based Environment for Generating Yield Estimation Maps From Apple Orchards Using UAV Imagery and a Deep Learning Technique

**DOI:** 10.3389/fpls.2020.01086

**Published:** 2020-07-15

**Authors:** Orly Enrique Apolo-Apolo, Manuel Pérez-Ruiz, Jorge Martínez-Guanter, João Valente

**Affiliations:** ^1^ Área de Ingeniería Agroforestal, Dpto. de Ingeniería Aeroespacial y Mecánica de Fluidos, Universidad de Sevilla, Sevilla, Spain; ^2^ Information Technology Group, Wageningen University & Research, Wageningen, Netherlands

**Keywords:** deep learning, apple, yield map, Google Colab, photogrammetry, fruit

## Abstract

Farmers require accurate yield estimates, since they are key to predicting the volume of stock needed at supermarkets and to organizing harvesting operations. In many cases, the yield is visually estimated by the crop producer, but this approach is not accurate or time efficient. This study presents a rapid sensing and yield estimation scheme using off-the-shelf aerial imagery and deep learning. A Region-Convolutional Neural Network was trained to detect and count the number of apple fruit on individual trees located on the orthomosaic built from images taken by the unmanned aerial vehicle (UAV). The results obtained with the proposed approach were compared with apple counts made *in situ* by an agrotechnician, and an *R^2^* value of 0.86 was acquired (MAE: 10.35 and RMSE: 13.56). As only parts of the tree fruits were visible in the top-view images, linear regression was used to estimate the number of total apples on each tree. An *R^2^* value of 0.80 (MAE: 128.56 and RMSE: 130.56) was obtained. With the number of fruits detected and tree coordinates two shapefile using Python script in Google Colab were generated. With the previous information two yield maps were displayed: one with information per tree and another with information per tree row. We are confident that these results will help to maximize the crop producers' outputs *via* optimized orchard management.

## Introduction

The successful management of modern, high-density apple orchards depends on the ability to improve processes such as planting, cultivation, harvesting, and the optimization of fruit commercialization (González-Araya et al., 2015). The efficient management of these tasks, where harvesting and fruit processing are considered high-cost, high value-added operations, is key for producers (Silwal et al., 2016). Consequently, an accurate yield estimation is crucial for the stakeholders (apple growers and sellers), since this information can significantly contribute to their decisions-making process (Gongal et al., 2015; Tian et al., 2019b).

The traditional management of agricultural crops has been inherently subjective and based on past experience, manually counting, and historical data collected by farmers (Rahnemoonfar and Sheppard, 2017). These methods can be inaccurate, subjected to bias, and inefficient, since they do not reflect the yield distribution across the orchard, especially in orchards with a high spatial variability (Aggelopooulou et al., 2013; Bargoti and Underwood, 2017). Currently, with the breakthrough of new agricultural technologies, many farm tasks are becoming automated, and researchers and companies have carried out studies based on artificial intelligence algorithms which automatically learns decision rules from data (Abiodun et al., 2018). A particular success has been the use of deep learning (DL) and, in particular, the development and application of a branch of these techniques known as Convolutional Neural Networks (CNN). These complex algorithms use images tagged by technicians or crop experts as inputs. These are laid out through various convolutional filters that activate image features to generate a trained model. As reviewed by other authors, the use of this models makes it possible to simplify and automate some of the analytical tasks in the agricultural domain (Kamilaris et al., 2017; Jha et al., 2019). Therefore, for example, a model for detecting and mapping every piece of fruit in a commercial mango orchard was proposed by Stein et al. (2016). The fruits were detected using a model based on Faster R-CNN. Koirala et al. (2019) tested several deep learning architectures to detect mango fruits on RGB images taken from a terrestrial vehicle during the night. Additionally, a method where synthetic images were used to train the model and tested on actual images was suggested by Rahnemoonfar and Sheppard (2017). Moreover, Fu et al. (2018) presented a system to detect kiwifruit in field images under different lighting conditions.

In the specific case of apple orchards, works employing different approaches have been explored by many researchers. Tian et al. (2019b) developed an improved model for apple detection during different growth stages. An object detection architecture named Yolo-V3 was used, and images with different light conditions at ground level were obtained. The pre-harvest yield mapping of apple orchards using segmentation techniques was suggested by Roy et al. (2019). Their contribution was the use of two clustering methods: semi-supervised (to separate the apple pixels from others in the input images) and unsupervised (to automatically identify the apples). Fruit size was estimated by Gongal et al. (2018) using the 3D coordinates of pixels from images taken by a 3D-camera as a tool for harvesting robots. A fine-tuned model for apple flower detection was deployed by Dias et al. (2018). The high accuracy of these approaches opened the door for the possible integration of these models into complex automated decision-making systems in the future. Nevertheless, existing methods can be improved, since many of the images used were taken by terrestrial vehicles and at ground level. This means that labor remains an inefficient aspect, since specific platforms are required for the taking of images, which constitutes a time-consuming task and can accentuate soil compaction problems.

Unmanned aerial vehicles (UAVs) are currently modernizing the farming industry by helping farmers to monitor their crops in a more timely manner (Mogili and Deepak, 2018). These aerial platforms usually mount high-resolution cameras that are capable of acquiring quality images (thermal, spectral, multispectral, or RGB-visible images), which can be used for various kinds of analysis (Maes and Steppe, 2019). Moreover, these vehicles can integrate an RTK-GNSS system for precise real-time positioning allowing the generation of crop maps with a centimeter-level accuracy at the field level (Chlingaryan et al., 2018). A general method used for creating crop maps is based on the structure from motion (SfM) algorithm (Turner et al., 2012). This algorithm selects important features known as keypoints from individual images to build a georeferenced orthomosaic (Anders et al., 2019). However, despite its suitability, producing these kinds of maps requires costly commercial software, a powerful computer, and multiple supervised steps to generate the new composite images (Torres-Sánchez et al., 2018). According to the literature reviewed, the most common types of photogrammetry software under private licenses used for this purpose are: Pix4D® (www.pix4d.com), AgisSoft PhotoScan® (www.agisoft.com), and Photomodeler® (www.photomodeler.com). However, in the recent years, the emergence of platforms, such as Docker (www.docker.com) or Django (www.djangoproject.com), have opened up the possibility of implementing the SfM algorithm in the cloud and developing open-source tools that are affordable for everyone at both professional and educational levels.

On the other hand, many of the remote sensing applications in agriculture are based on using Geographical Information Systems (GIS) to bring value to the farmers (Machwitz et al., 2019; Maes and Steppe, 2019). These tools allow us to prepare and manage agricultural georeferenced data and build geospatial snapshots of cropland from remote sensors mounted on both aerial and terrestrial platforms (Sharma et al., 2018). The information generated enables the automation of field operations, the reduction of costs, and maximization, acting as a steward of the land (Kaloxylos et al., 2012). Until a few years ago, the most popular types of software for GIS applications were Quantum GIS (www.qgis.org) and Esri's ArcGIS (www.arcgis.com). The first is open-source but the other needs a commercial license (Duarte et al., 2017). The use of this software requires the user to have a basic knowledge of how to work and interpret the data contained in raster and shape files (the most common files used in GIS), although it is not always an easy task, especially for farmers (Abdelrahman et al., 2016). In recent years, a collection of open-source GIS libraries that work with Python language have been developed and made available to the general public (Gillies, 2007; Jordahl, 2014; Rapiński and Zinkiewicz, 2019). Examples of this type of library are GeoPandas (www.geopandas.org), GeoServer (www.geoserver.org), and Qhull (www.qhull.org), among others. At the same time, the development of platforms such as Google Colaboratory (www.colab.research.google.com), a cloud service based on Jupyter Notebooks, which allows the integration of deep learning models and GIS tools in a simple python script, has occurred (Carneiro et al., 2018; Bisong, 2019). This provides the opportunity to develop geospatial analysis tools that can be readily integrated into web platforms, allowing their adoption by farmers.

Based on the above, it can be asserted that the high cost of data collection and the difficulty of interpretation currently prevents farmers from implementing data-driven agriculture (Thompson et al., 2019). With specific regard to yield mapping in apple orchards, based on the detection of the number of fruits, although the proposed methods have shown promising results and a high accuracy, they do not provide a final product with a high potential to be exploited by the farmers. Additionally, most of them use ground-level platforms that may increase the data collection time and hinder their application in large agricultural areas.

Therefore, the objectives of this project were the following: (1) exploring the feasibility of yield estimation by detecting apple fruits on images taken by a UAV; (2) training and testing a model based on CNNs to automatically detect apple fruits, with the aim of making the weights and models used for apple detection available for the general public; and (3) building an apple tree variable yield map for each tree and one with information per each tree row.

## Materials and Methods

### Location and Imagery Acquisition

This study was undertaken during the 2018 and 2019 seasons in an orchard fields of apple (*Malus x Dornestica* Borkh. cv ‘Elstar') in Randwijk (latitude: 51°56'18.5”N; longitude: 5°42'24.8”E) near Wageningen (The Netherlands). The crop field had 0.47 ha with 592 trees allocated in 14 rows with approximately 41 trees in each row and a pollinator tree every 10 m. The average tree height was 3 m with a tree spacing was 3 × 1 m (inter-row and intra-row), rows were NW-SE oriented, and the crop management tasks (fertilization, thinning, pruning, etc.) were performed following the conventional farm practices.

**Figure 1:**
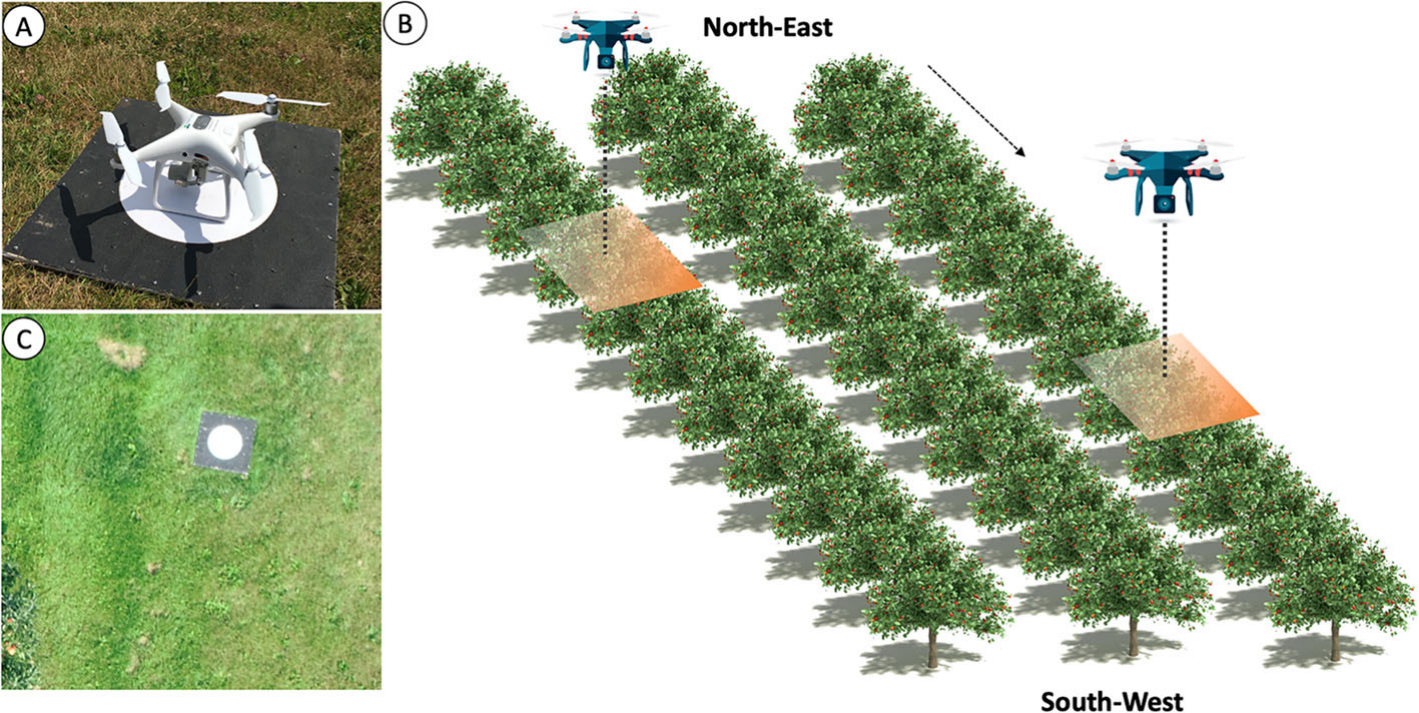
Field test flight design and equipment used **(A)** unmanned aerial vehicle (UAV; DJI Phantom 4) used during the flights, over one of the ground control points to associate projection coordinates with locations on the images; **(B)** workflow for UAV image acquisition; and **(C)** an image of an apple tree in the field.

The UAV platform employed to take the pictures was a DJI Phantom 4 Pro (DJI Technology Co., Ltd., Shenzhen, China) at a set flying altitude of 10 m (**Figure 1A**). The onboard camera had a 1/2.3'' CMOS sensor (with an effective pixel count of 20M), a lens FOV of 84°, a focal length of 8.8 mm, a focal ratio of f/4.5, and a focus to infinity. This UAV was equipped with dual-band satellite positioning (GPS and GLONASS), which provided a sub-meter precision location.

A grid-shaped flight plan was designed using the DJI Ground Station Pro (DJI Technology Co., Ltd., Shenzhen, China) iPad application, which allowed us to control or plan automatic flights for DJI aircrafts (Chen et al., 2019). The flights, in the two seasons (2018 and 2019), to take the pictures were made 2 days before the first harvest (40%). It was a sunny day with low wind speed. A total of 806 pictures at 15 m above the ground were taken in a nadiral view (vertically downward at 90°) (**Figure 1B**). The image resolution was set to 5,472 × 3,648 pixels (JPG format). A total of 354 images taken in 2019 were used to build the dataset for training the CNN, while the rest (taken in 2018) were used for creating the visible orthomosaic. These latter were obtained with a forward overlap of 85% and a sideway overlap of 75%. The UAV flight made in 2018 had to be made over a portion of the trees because the rest of the field had already been harvested by the farmer.

Five ground control points (GCPs) were established during each flight as an indirect georeferencing of UAV images and for an accuracy assessment of the orthomosaic obtained (**Figure 1C**). The precise locations of the GCP (black and white targets) were obtained using a Topcon RTK GNSS equipment with an accuracy below 2.5 cm. A total of 452 pictures were used for orthomosaic creation.

### Ground Truth Acquisition for Yield Estimation

**Figure 2:**
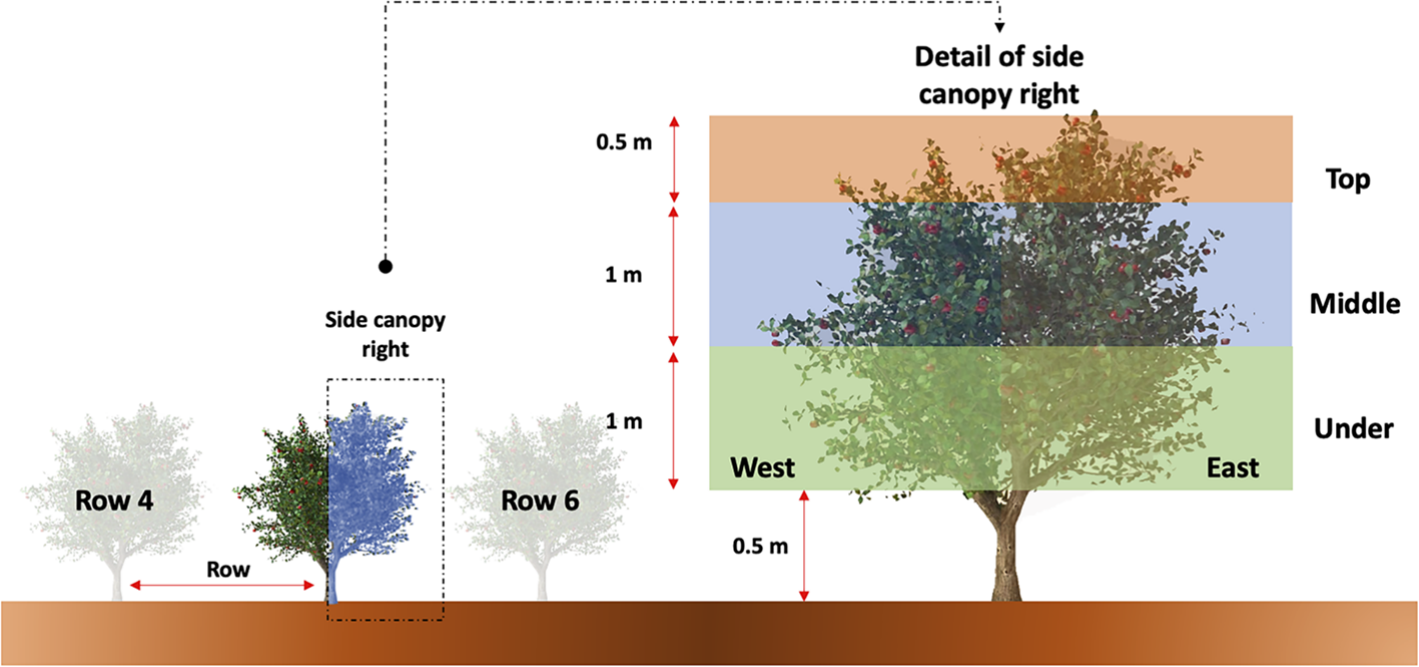
Detailed illustration of apple tree canopy architecture division for visual counting of fruit from both the right and left sides of the row.

According to Moltó et al. (1992) and Jiménez et al. (2000), only approximately 60–70% of crop production is visible from the outside of a tree; here lies the complexity of yield estimation, as not all existing fruits can be detected with only external images of the tree. Moreover, previous studies have been based on ground-level observations on both sides of the tree canopy. However, zenithal pictures shown only a fraction of the total fruits, making it a challenge to generate complex models for yield estimation in this type of study (Chen et al., 2019). On this basis, a previous step in this research was to check the percentage of fruit visible from the aerial pictures. At the same time as the pictures to build the orthomosaic were taken by the UAV before harvesting, a representative sample of 19 trees was randomly selected from row 5 of the crop field. We assumed the number of fruits by row remain consistent based on historical data provide by the farmer. The tree architecture was divided according to **Figure 2**. Then, visual counting of the fruit was conducted on each side (right and left) of the tree, and the data were collected in a Microsoft Excel (Version 16.37) file. For avoiding duplicated counting of the fruits, a plastic tape was used to delimit the areas of interest.

Later, the apple fruits in all trees were hand-harvested and weighed to give an average weight in kilograms per meter (kg/m) of fruit per row. The collection of fruit was conducted in three stages, since the market demand for fresh fruit is variable over the time during a harvest season (Lötze and Bergh, 2004). Moreover, farmers tend to choose the best moment to be able to find a good price for their product.

### Orthomosaic Construction and Data Pre-Processing for Yield Map Estimation

**Figure 3:**
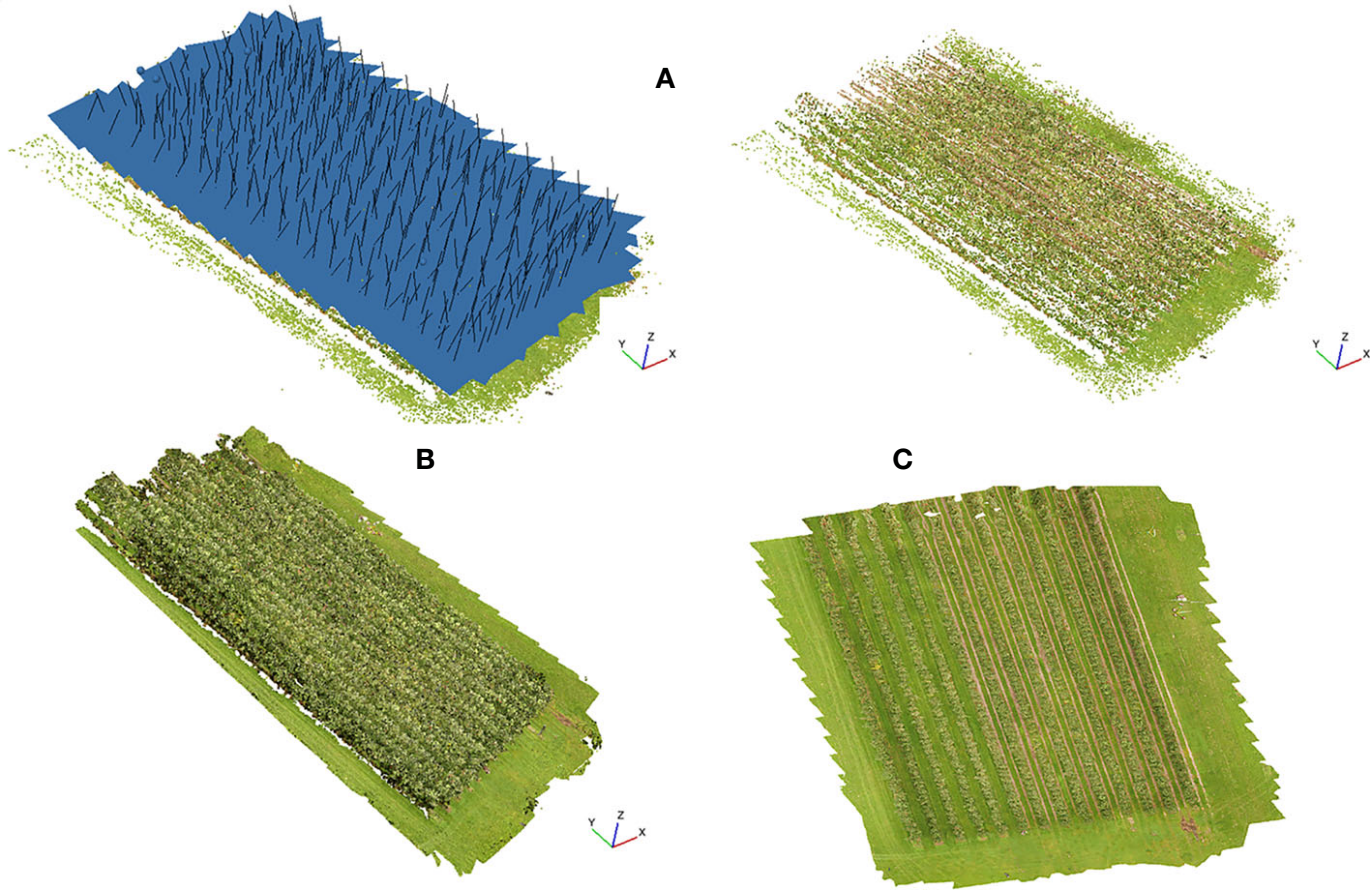
Workflow to build an orthomosaic using Agisoft PhotoScan: **(A)** photo alignment and 3D sparse point cloud (each blue square represents the estimated viewpoint of the input images). **(B)** dense point cloud, and **(C)** orthomosaic.

A total of 452 images were used to build the orthomosaic [an aerial image of an area, composed of multiple images stitched together using photogrammetry which has been geometrically corrected (Chen et al., 2019)]. The imagery was automatically processed using Agisoft PhotoScan Professional 1.2.3 software (Agisoft LCC, St. Petersburg, Russia). Following the software recommendations, the first step was to “Align Photos” with the “High” accuracy set up. This option uses the original resolution of images to generate a sparse 3D point cloud with a low resolution as a necessary first step towards building the orthomosaic. After that, GCPs were manually located in each image. This process is necessary, as, despite of the images taken by the UAV being geotagged using the onboard GNSS receiver, the accuracy of this sensor is low. Then, a 3D dense point cloud (110449395 points) with a “High” accuracy was generated in a previous step to build the final raster file (**Figure 3**). Finally, the orthomosaic in the coordinate system WGS 84 (EPSG: 4326) was exported as a GeoTIFF file with 4.18 mm/pixel to be further used in fruit detection and to build the yield map based on the number of fruits detected.

Currently, apple orchards are being planted using advanced machinery that records GNSS coordinates of each tree in a standard vectorial format (shapefile) of the GIS. These files allow storage spatial information and vector operations with other files, such as raster files (Oliver, 2013; Maes and Steppe, 2019). On this basis, a python script was developed to create a circular mask (1-meter diameter) with the coordinates of each tree. The output's script was an individual shapefile for each tree avoiding the edges of the canopy. Then, the orthomosaic was cropped using these shapefiles, and a TIF file was obtained for each tree as an output. Finally, each TIF file was tested using the Faster R-CNN model to count apple fruits. Considering the number of fruits detected and taking into account and the distribution of fruits on the structure of the apple canopy, a yield map estimation was created using Qgid (3.12).

### Building and Labeling Image Datasets for Apple Fruit Detection

Dataset size plays a critical role in making DL models successful. A model without sufficient and representative training data is not able to learn the fundamental discriminative patterns required to carry out robust detection of fruits (Sonka et al., 1993). The features of apples on the trees may dramatically diverge (e.g., green fruits, fruits of different sizes, fruits occluded by branches and leaves, and overlapping fruits). Moreover, the images might suffer from distortions, especially those generated by outdoor light conditions and the rolling shutter effect (Chen et al., 2019).

**Figure 4:**
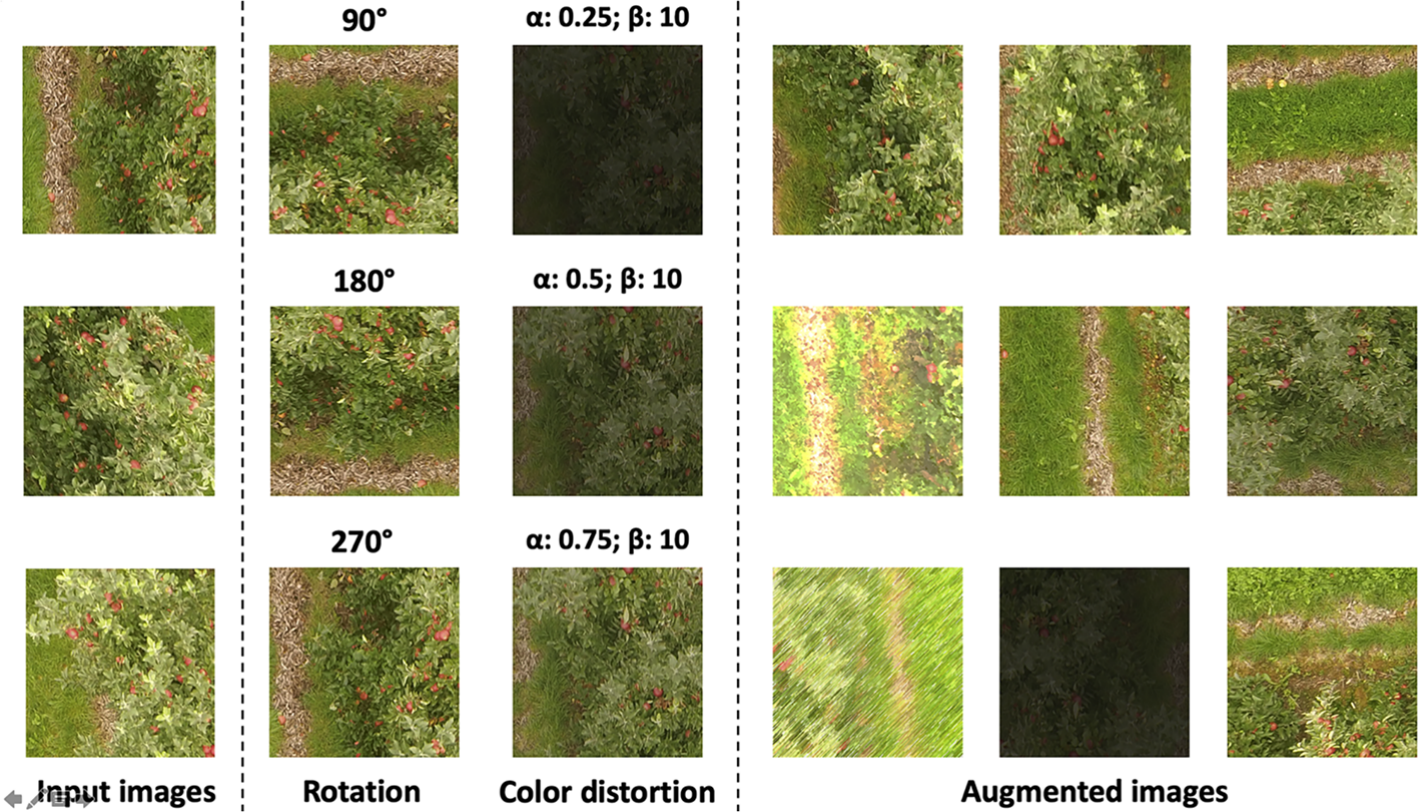
Dataset augmentation process. Original images were rotated 90, 180, and 270 degrees and color distortion ware setting changing both α and β values.

The set of processes carried out by a CNN requires images with an appropriate resolution, since high-quality images increase the computational resources needed (Lecun et al., 2015; Chollet, 2017). Therefore, the images taken by the UAV in this study were cropped to produce smaller images with a resolution of 416 × 416 px without applying any resizing process. As a result, a preliminary sample of 1,000 images was selected to train the model. Additionally, in order to achieve a high accuracy and avoid overfitting problems, data augmentation techniques were applied (Krizhevsky et al., 2012; Simonyan and Zisserman, 2014). Data augmentation is a common technique used to transform pictures based on rotation, changing color channels, and the addition of filters among others. In this paper, images were rotated by 90, 180, and 270 degrees. The contrast and brightness were changed by varying α and β values responsible for the color difference settings using a Python script developed by the authors (**Figure 4**). Consequently, a dataset containing a total of 3,000 pictures was used to train the CNN.

**Figure 5:**
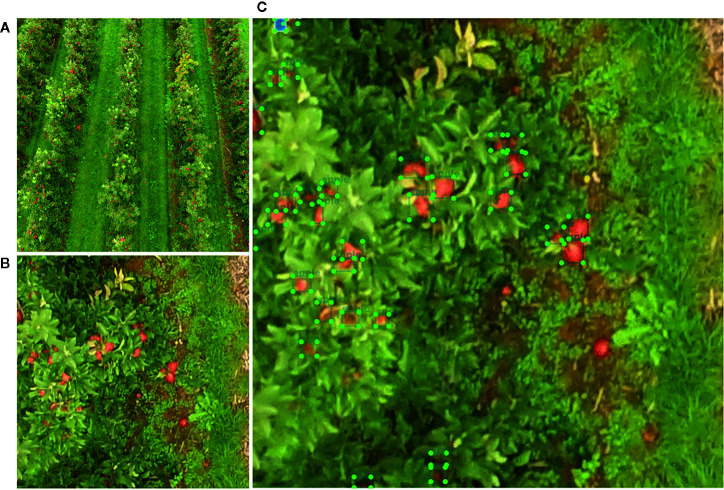
Labeling process used to annotate pictures: **(A)** original picture taken by UAV; **(B)** picture with 416 × 416 px resolution; and **(C)** picture with each one of the bounding boxes.

As suggested by Rahnemoonfar and Sheppard (2017), CNN requires a huge amount of annotated pictures with the coordinates of each fruit on the images from the training dataset. In this project, a free and open-source labeling tool called LabelImg (v1.8.3) was used (Tzutalin, 2015). The process was done manually and very carefully to prevent mislabeling or occlusion, since, due to the nature of fruit trees, many of them were completely occluded by others or even attached to each other (**Figure 5**). Once all fruits had been labeled with a bounding box, an Extensible Markup Language (XML) file in PASCAL Visual Object Classes (VOC) format was generated.

Once the labeling process was complete, the configuration details for the model and labels were implemented in the TensorFlow API (www.tensorflow.org). Due to CNN's high demand for hardware and GPU resources, Google Colaboratory (also known as Colab) offered by Google was used to implement and train the model. Colab, a cloud service based on Jupyter Notebooks, provides a free single 12GB NVIDIA Tesla K80 GPU that can be continuously used for up to 12 h. The advantage of this particular tool lies in the fact that its access is completely free and open-source. It also allowed us to work in the same work space with geospatial data and DL algorithms. We consider this platform to be a powerful tool that may in the future play a determining role in research and education with aggregated data and expert decision-making systems based on georeferenced data and ML (Machine Learning) algorithms.

For the local computing processes, a MacBook Pro laptop (MacOs High Sierra 10.13.4) with a 2.5 GHz Intel Core i7 processor, 16 GB of RAM, and Graphics AMD Radeon R9 M370X 2048 MB Intel Iris Pro 1536 MB was used. The Open-Source Computer Vision (OpenCV) library (http://opencv.org/), which includes several hundred computer vision algorithms, was used to process images (Rosebrock, 2016). The Keras (Chollet, 2017) open-source library was used in combination with TensorFlow backend tools to build and deploy the DL architecture.

### Fine-Tuning and Training of the Faster-RCNN

**Figure 6:**
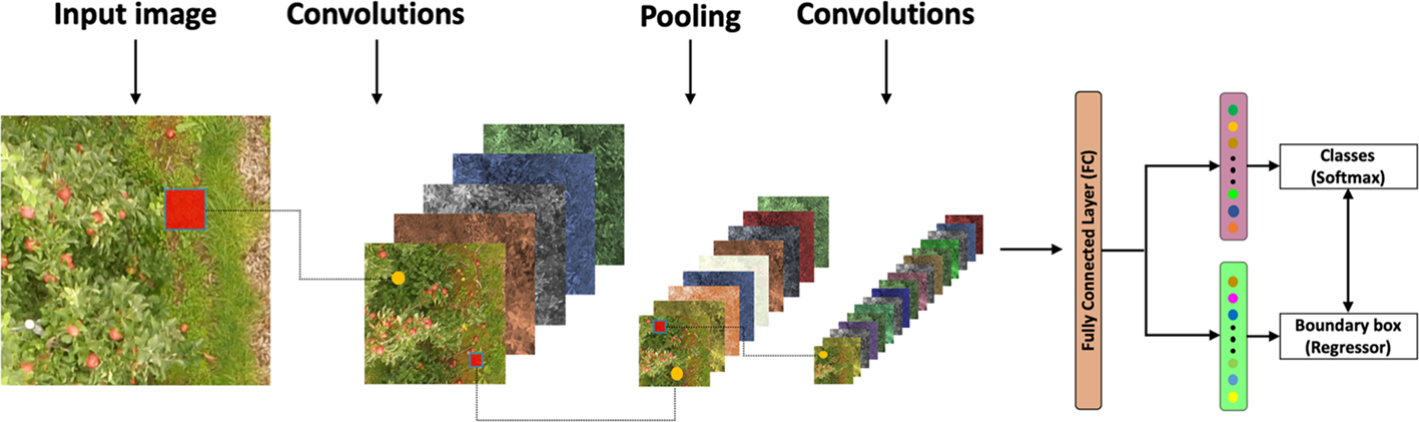
General architecture of a convolutional neural network.

Convolutional neural networks have been proven to be powerful visual models that use complex data as inputs that are capable of conducting automated fruit counting in the images. These algorithms consider an image as a matrix of pixels whose size (kernel) is (height × width × depth), where the depth is the number of image channels (3 for our RGB crop images). Hidden layers with a hierarchical structure (Lecun et al., 2015) are the main components of a CNN; the first layers can detect lines, corners, and simple shapes, whereas deeper layers can recognize complex shapes (Rosebrock, 2018). A common CNN architecture consists of several convolutional blocks (composed of convolutional layer + pooling layers + non-linearity) and one or more fully connected layers (**Figure 6**). Feature extraction, non-linearity operations, and dimension reduction were performed with this common architecture. Additionally, a fully connected layer was used to classify data from images (Guo et al., 2016), while a softmax function assigned the probability of belonging to the class (apple).

Despite the advances in computational processes and the available power offered by the graphics processing unit (GPU), training a neural network from scratch is still highly computationally expensive and requires large datasets for learning (Patrício and Rieder, 2018). To overcome these obstacles, a method named transfer learning (Gu et al., 2018) was used. The main objective of this procedure is to transfer the knowledge from one model trained on large datasets, such as ImageNet (Gopalakrishnan et al., 2017), to another model to solve a specific task (Talukdar et al., 2018). Several popular pretrained networks using transfer learning, such as VGG-16, ResNet 50, DeepNet, and AlexNet Inception V2, are described in the literature (Rosebrock, 2018).

**Figure 7:**
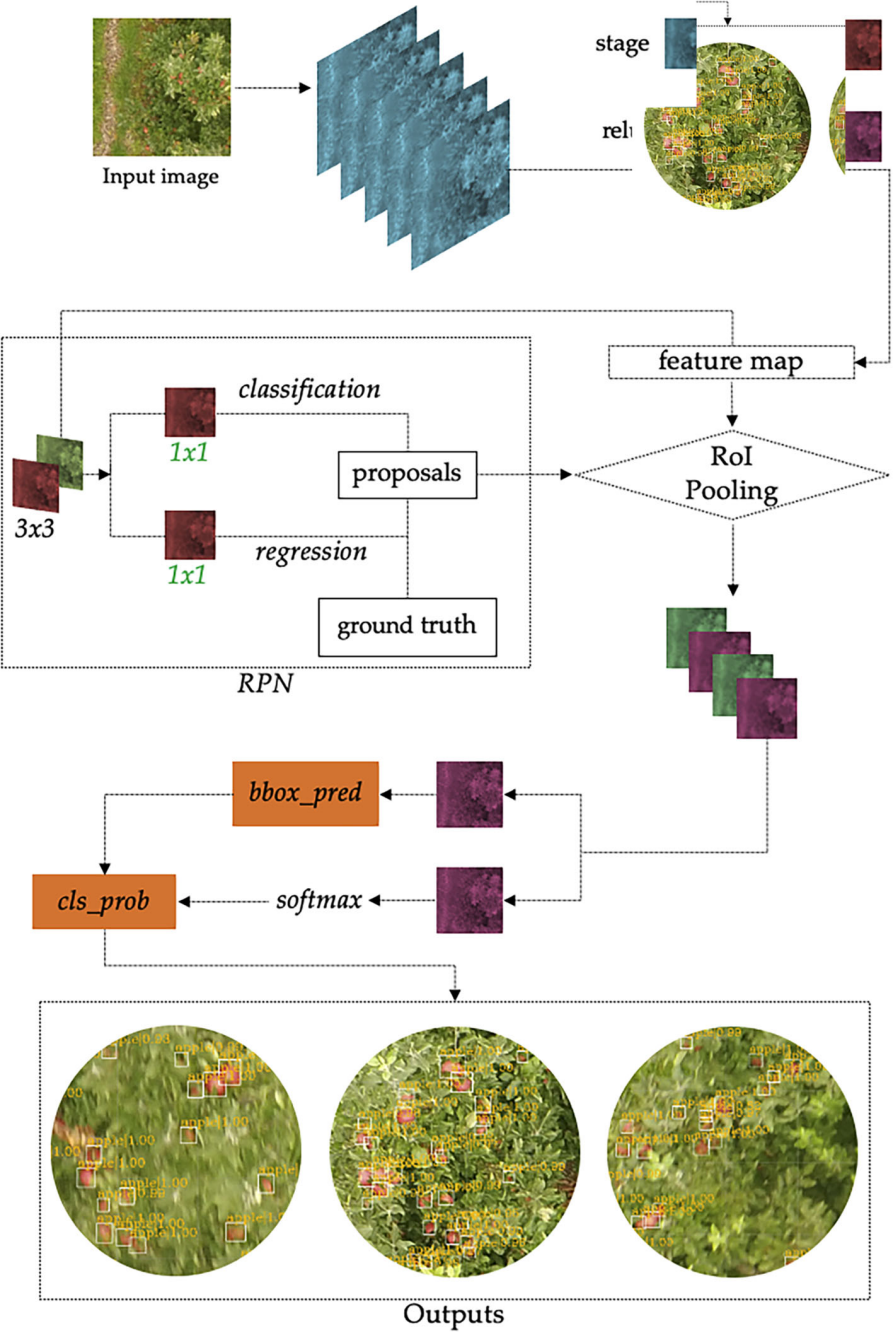
The architecture of Faster R-CNN. “conv” represents the convolutional layer, the “relu” represents the activation function, and the “fc layer” represents the fully connected layer. The network outputs intermediate layers of the same size in the same “stage.” “bbox_pred” represents the position offset of the object, “cls_prob” represents the probability of the category, and the outputs show the fruits detected.

The Faster R-CNN model was selected, since this network can use several architectures, such as ResNet, Inception, and Atrous, and thus increase the efficiency and precision of fruit detection (Dias et al., 2018). In this study, the Faster R-CNN Inception Resnet V2 Atrous Coco (Ren et al., 2017) model with a TensorFlow object detection application programming interface (API) was used. TensorFlow is an open-source software library for numerical computations (Kamilaris and Prenafeta-Boldú, 2018) and was used because of its flexibility and the ability to deploy network computations in multiple central processing units (CPUs), GPUs, and servers. The model comprises three steps, with an apple tree image as the input. Faster R-CNN extracts feature maps from the image using a CNN and then passes these maps through a region proposal network (RPN), which returns object proposals (Rosebrock, 2018). Finally, these maps are classified, and the bounding boxes enclosing the apple fruits are predicted (**Figure 7**).

The model was trained for 6 h, until the loss function reached the value of 0.06. This function allowed an accurate quantification of the model to ensure correct classification of the apples in our dataset (Kamilaris and Prenafeta-Boldú, 2018). The batch size (the parameter that defines the number of samples, which are images in this case, that will be propagated through the CNN) was two images in each step. The learning rate (a hyperparameter which determines the learning speed of the new information over the old) was 0.001.

### Statistical Analyses

To evaluate the accuracy of the trained model, 20 randomly selected pictures cropped from the orthomosaic randomly selected were used. The total number of fruits per picture (Nfp) was manually counted using the Photoshop count tool (Adobe Systems Inc., San Jose, United States), as suggested by Payne et al. (2014). Consequently, with this data, the precision (P, Eqn. 1), recall (R, Eqn. 2), F1score (Eqn. 3), and Accuracy (A, Eqn. 4) were used as the evaluation metrics for fruit detection (Rosebrock, 2018). These model evaluation metrics are defined as follows:

Eqn. 1 $$Precision\;\left(P\right)=\;\frac{TP}{TP+FP}$$

Eqn. 2 $$Recall\;\left(R\right)=\;\frac{TP}{TP+FN}$$

Eqn. 3 $$F1score=2x\frac{PxR}{P+R}$$

Eqn. 4 $$Accuracy\;\left(A\right)=\;\frac{TP}{Nfp}$$

where TP corresponds to true positives, i.e., when the algorithm correctly detects a fruit with a bounding box; FP indicates false positives, i.e., when a box is computed in a location where a fruit is not located; and FN denotes false negatives, i.e., when a target fruit is not detected.

Linear regressions were used for comparisons of the number of fruits counted visually (in the field and on the pictures) and the number of fruits harvested. The analysis was performed with RStudio® (http://www.rstudio.com). A comparison of visually counted fruits and harvested fruits was performed using the Mean Absolute Error (MAE, Eqn. 5) and the Root Mean Square Error (RMSE, Eqn. 6):

Eqn. 5 $$MAE=\frac{1}{n}\sum_{t=1}^{n}\left|A_{t}-F_{t}\right|$$

Eqn. 6 $$RMSE=\sqrt{\frac{\sum_{t=1}^{n}{\left(A_{t}-F_{t}\right)}^{2}}{n}}$$

where n refers to the number of compared values, A_t_ is the actual observed value, and F_f_ is the forecast value.

## Results

### Distribution of Fruits in an Apple Orchard Canopy

The distribution of the fruits inside an apple canopy tree can be strongly variable. It depends on several factors, such as the tree height, the effect of row orientation on daily light absorption, and the apple cultivar planted in the field, among others (Gongal et al., 2018). **Table 1** shows that the largest amount of fruits was found between the middle and underside of the tree. This could be explained by the canopy architecture, since, on the top of the tree, generally, there is a smaller number of branches (Willaume et al., 2004). Furthermore, farmers tend to prune apple trees to concentrate the majority of the fruits in the middle and underside of the tree. This fruit distribution makes it much easier for the fruit picking operator during the harvesting process (Brendon et al., 2019).

**Table 1:** Data on the trees randomly selected in the crop field.

Id	Nfcvt	Nfcvm	Nfcvun	Total of fruits	Top (%)	Middle (%)	Underside (%)
1	88	95	92	275	32.00	34.55	33.45
5	85	101	99	285	29.82	35.44	34.74
9	66	77	95	238	27.73	32.35	39.92
14	83	68	77	228	36.40	29.82	33.77
17	56	75	82	213	26.29	35.21	38.50
24	51	93	82	226	22.57	41.15	36.28
25	86	87	70	243	35.39	35.80	28.81
27	77	107	124	308	25.00	34.74	40.26
32	53	78	125	256	20.70	30.47	48.83
33	88	117	64	269	32.71	43.49	23.79
34	56	89	107	252	22.22	35.32	42.46
43	35	202	82	319	10.97	63.32	25.71
48	80	117	70	267	29.96	43.82	26.22
50	90	95	72	257	35.02	36.96	28.02
51	63	44	68	175	36.00	25.14	38.86
56	77	84	78	239	32.22	35.15	32.64
57	48	128	97	273	17.58	46.89	35.53
59	69	88	98	255	27.06	34.51	38.43
62	52	110	108	270	19.26	40.74	40.00
**Average**	68.58	97.63	88.95	255.16	27.31	37.63	35.06

Id, number of the tree in the field; Nfcvt, Number of fruits visually counted on the top of the tree; Nfcvn, Number of fruits visually counted in the middle of the tree; Nfcvun, Number of fruits visually counted in the lowest part of the tree; (%), percentage values for each part of the tree regarding the total.

It can also be observed that each apple tree contained between 175 and 308 fruits, with an average of 255. On the other hand, the percentage of fruits on the top of the tree had an average value of 27.31%. Hence, it must be realized that only a part of this percentage of fruits was detected on the images obtained with the UAV.

**Figure 8:**
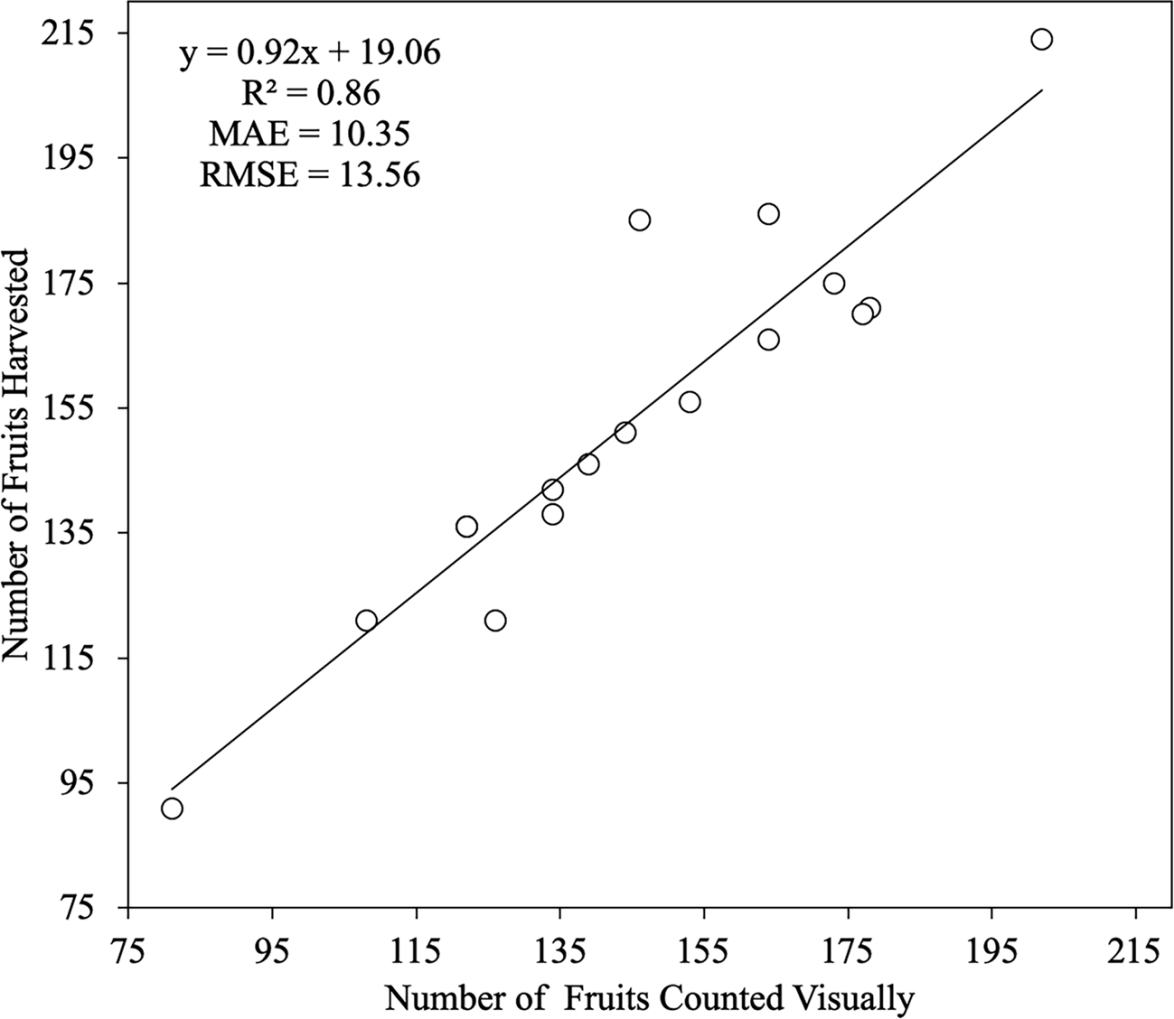
Linear regression between both the Number of Fruits Counted Visually in the field and the Number of Fruits Harvested. The number of trees analyzed was n = 19. The straight line represents the best-fit linear regression (p < 0.001).

When visual counts of fruits are made before harvesting, the total number of them can be affected by many factors. The main reasons for this are natural fruit drop and biotic and abiotic factors. Another reason it may be due to visual errors committed by the staff devoted to counting the fruits (i.e., they may count the same fruit twice). In **Figure 8**, a linear regression between the number of fruits counted visually in the field and the number of fruits harvested is shown. An R^2^ value of 0.86 was obtained, which indicates a good correlation for both numerical variables. However, the MAE and RMSE values obtained were high, which indicates a bad model adjustment. The low consistency between the number of fruits counted visually and the fruits harvested is probably due to losses during the counting process when using a fruit grading machine. This kind of machine does not detect small-sized fruits; hence, the use of a manual process to count the fruit can improve model adjustment.

**Figure 9:**
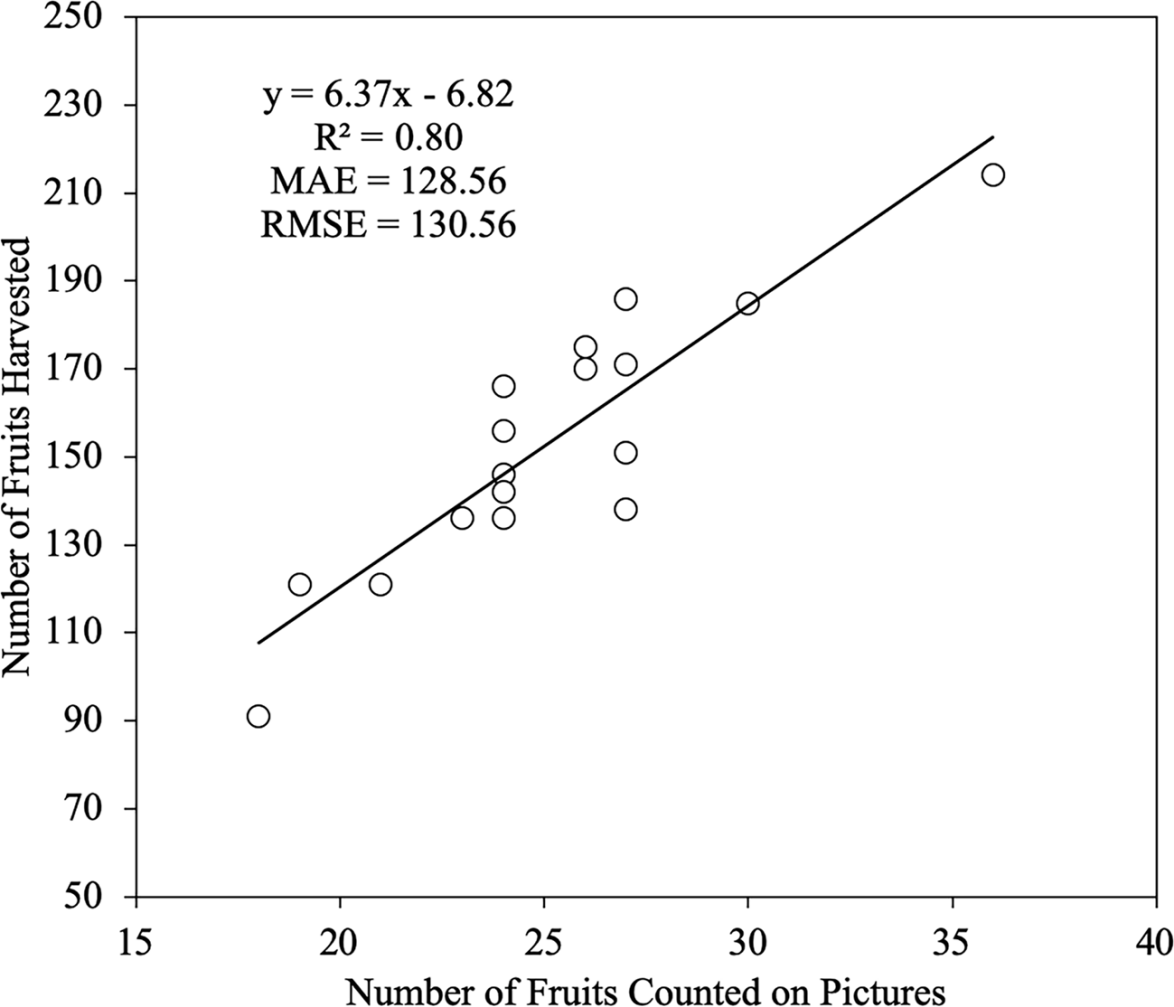
Linear regression between the Number of Fruits Counted on Pictures and the Number of Fruits Harvested. The number of trees analyzed was n = 19. The straight line represents the best-fit linear regression (p < 0.001).

The starting point was the premise that the human eye is the most accurate method for detecting fruit on the images (Rosebrock, 2018). In this sense, in **Figure 9**, a linear regression between the number of fruits counted on the image and the number of fruits harvested is displayed. An R^2^ value of 0.80 can be observed, which indicates a low correlation. As expected, the number of fruits detected in the images taken by the UAV is insufficient for estimating the rest of the fruits present in the canopy tree with traditional mathematical models. The results show that it is possible, although with a low accuracy, to make predictions of the total number of fruits in each tree using these kinds of images. The high values for MAE and RMSE suggest that, despite of all the fruits being detected using DL algorithms, the variability in the number of fruits harvested with respect to the number of detected fruits cannot be modeled well using standard linear regression.

### Distribution of Fruits in an Apple Orchard Canopy

**Figure 10:**
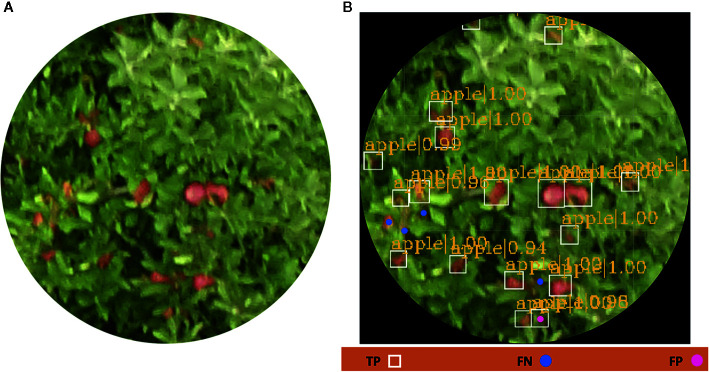
**(A)** Original RGB picture taken from an UAV and **(B)** apple fruits automatically detected with bounding boxes and their probabilities obtained by the Faster R-CNN model. The errors (TP, FP, and FN) are shown as a legend below the picture.

In **Figure 10**, the workflow from an input image until the fruits are detected is shown. Over each detected apple fruit, a blue bounding box with the probability of containing the fruits and a legend with TP, FP, and FN are shown in (**Figure 10B**). Based on the above, it was concluded that the 3,000 images tagged for apple detection were sufficient for explaining the wide variability in the data set, as the number of fruits detected was high. Apparently, the application of data augmentation helped to overcome the problems in object detection caused by illumination conditions, the distance to the fruit, and fruit clustering, among others, as suggested by Voulodimos et al. (2018).

In outdoor conditions, the model could not detect all fruits, but it was able to detect most of the visible fruits. It was observed that the pictures taken by an UAV suffered from notable changes, mainly due to unstructured light conditions and the camera's rolling shutter effect. Moreover, invisible fruits that are occluded by foliage or other fruits are the main challenge for DL models based on object detection (Kamilaris and Prenafeta-Boldú, 2018). Therefore, in **Table 2**, an analysis of the precision of fruit detection is presented. The values for each of the metrics used to assess the obtained results were greater than 90% in terms of precision (P). Similar results were obtained by Chen et al. (2019), although their results were slightly lower, probably due to the size of strawberry fruits, which are smaller than apple fruits. False positives were observed in the pictures that corresponded to immature fruits (fruits green), and where the brightness of sunlight was slightly greater, and in those pictures that suffered from rolling shutter. These results can be significantly improved by taking pictures several times throughout the day, as suggested by Fu et al. (2018), or by flying the UAV at a low speed. Finally, the F1-score exhibited values greater than 87%, indicating the high robustness of the trained model. On the other hand, with visual counting (Nfp), considered to be the most reliable method, an accuracy of 88.96% was obtained. The errors between visual counts and object detection were similar to those obtained by Neupane et al. (2019) when counting banana fruits. These results demonstrate that the use of simple data augmentation techniques such as picture rotation, filters, and transfer learning can facilitate the building of tools with a high potential for apple fruit detection.

**Table 2:** Fruit detection analyses for each of the pictures selected.

Picture	TP	FP	FN	P	R	F1	N_fp_	A
1	70	2	8	0.97	0.90	0.93	78	0.90
2	44	3	9	0.94	0.83	0.88	53	0.83
3	62	3	6	0.95	0.91	0.93	68	0.91
4	53	4	5	0.93	0.91	0.92	58	0.91
5	20	2	4	0.91	0.83	0.87	24	0.83
6	41	3	9	0.93	0.82	0.87	50	0.82
7	67	3	5	0.96	0.93	0.94	72	0.93
8	83	5	8	0.94	0.91	0.93	91	0.91
9	86	7	10	0.92	0.90	0.91	96	0.90
10	77	6	4	0.93	0.95	0.94	81	0.95
11	80	7	6	0.92	0.93	0.92	86	0.93
12	61	3	6	0.95	0.91	0.93	67	0.91
13	75	4	1	0.95	0.99	0.97	76	0.99
14	73	5	8	0.94	0.90	0.92	81	0.90
15	30	6	4	0.83	0.88	0.86	34	0.88
16	38	5	7	0.88	0.84	0.86	45	0.84
17	56	3	8	0.95	0.88	0.91	64	0.88
18	53	7	3	0.88	0.95	0.91	56	0.95
19	67	8	9	0.89	0.88	0.89	76	0.88
20	91	2	7	0.98	0.93	0.95	98	0.93
**Avg**	61.35	4.40	6.35	0.93	0.90	0.91	67.70	0.90

TP, True Positive; FP, False Positive; P, Precision; R, Recall; F1, F1score value; N_fp_, Number of fruits counted on pictures visually; A, Accuracy.

### Yield Map Creation

**Figure 11:**
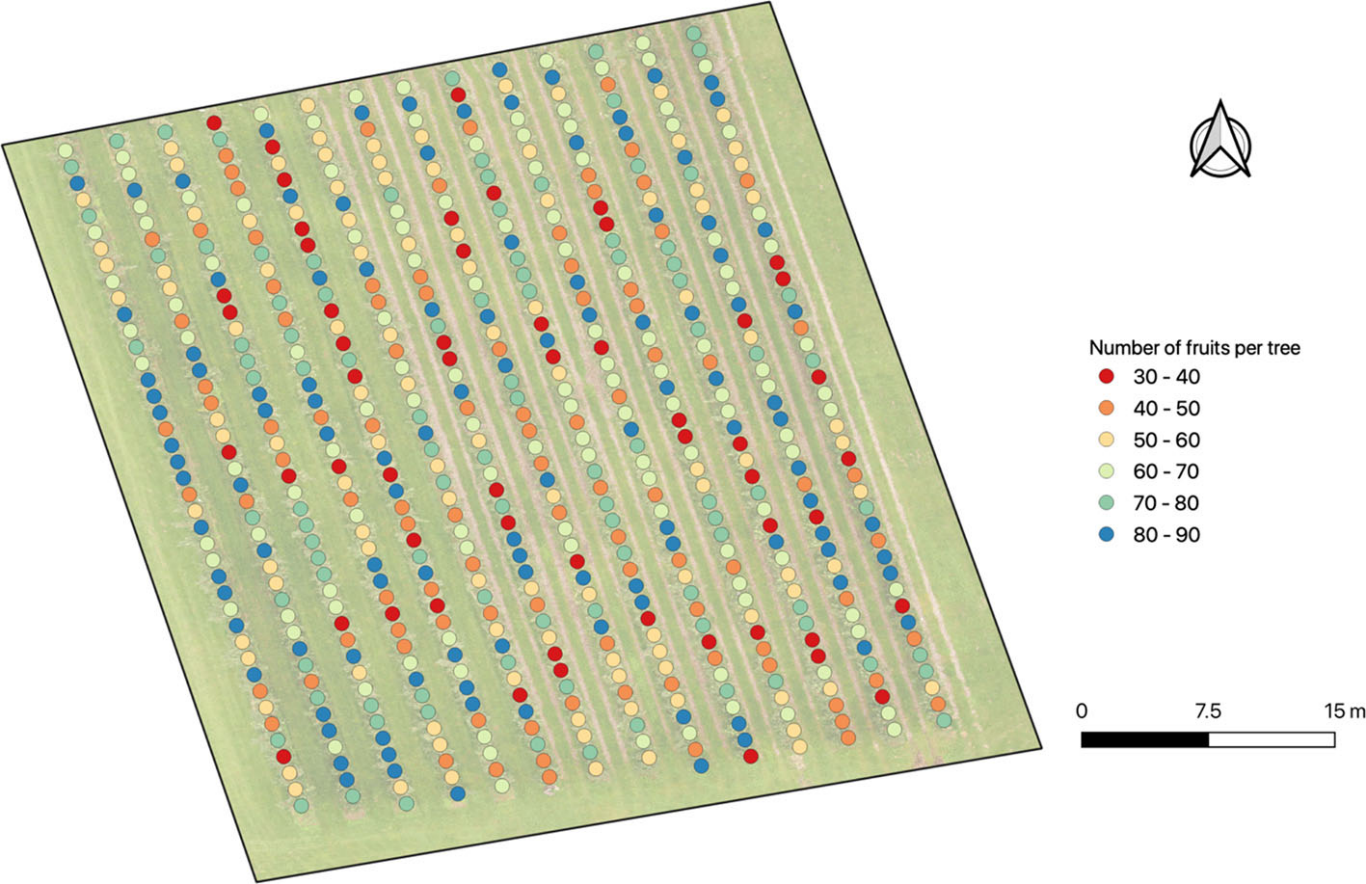
Apple yield map with the number of fruits per tree detected with the Faster R-CNN model trained.

As seen in the previous sections, a highly accurate estimation of the number of fruits per tree is not easy or straightforward. Nevertheless, it is possible to build an apple yield map as a tool to at least approximately determine the number of fruits in each tree of the crop field. This foreseen information could be useful for both farmers (to know the number of staff needed to be contracted) and contractors (to know the volume of production to be transported). In **Figure 11**, there is an apple yield map in which the number of fruits per tree detected is shown. It allows a visualization of the high spatial variability in the field, as well as the expected number of fruits per tree. It also may be affirmed that there is a low percentage (9.12%) of trees with a number of fruits between 30 and 40.

**Figure 12:**
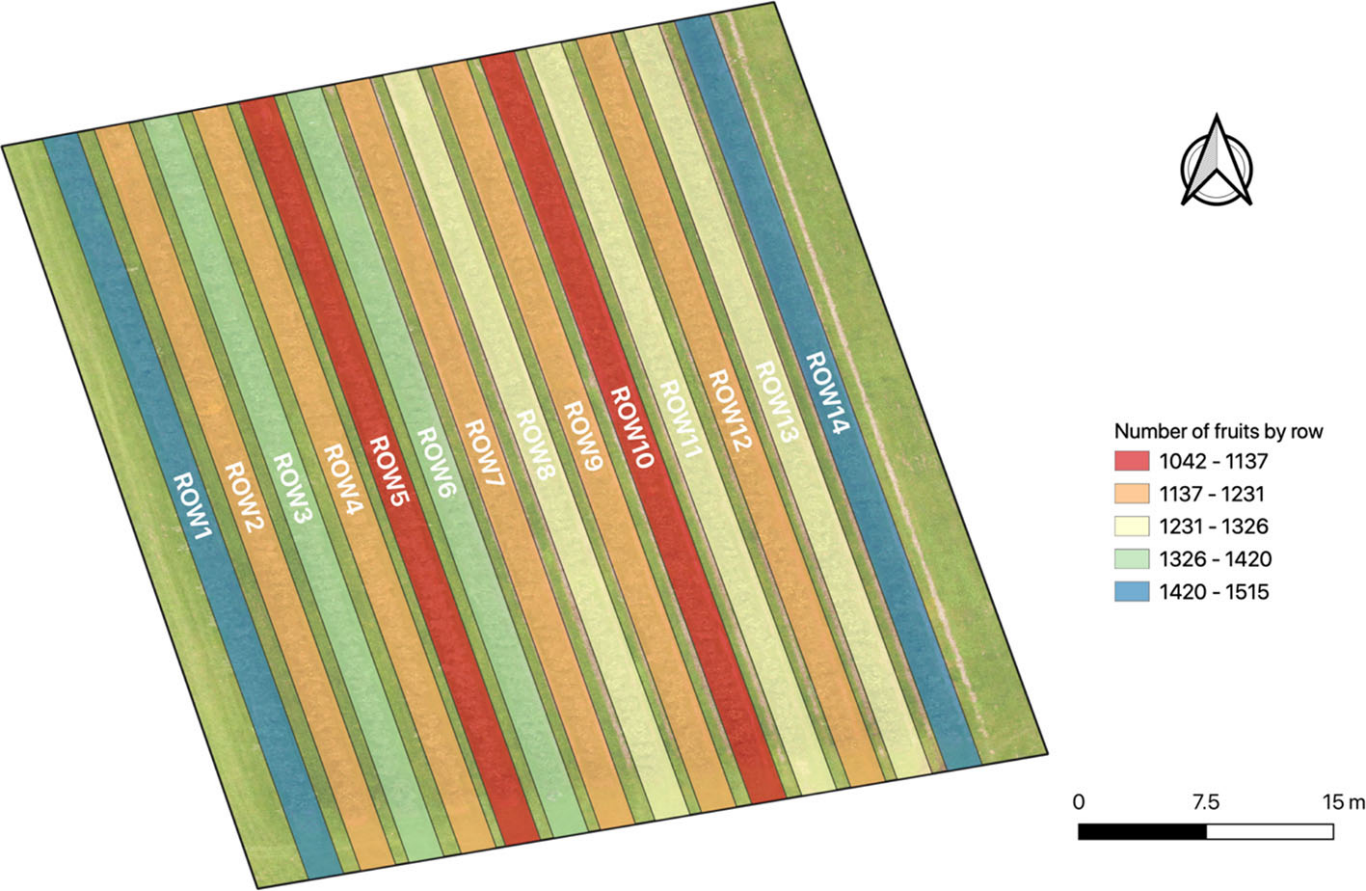
Apple yield map with the total number of fruits by row detected with the Faster R-CNN trained model.

Tree-level information can be useful, but it could be more interesting to have the same information for each row of the crop field. In **Figure 12**, a more actionable apple yield map with the total number of fruits for each row is shown. The results show that row 5 and row 10 contain less fruits in their trees. Meanwhile, row 1 and 14 are the rows with the greatest volumes of fruits. The rest of the rows have a similar number of fruits.

## Discussion

### Computational Time Required

According to Torres-Sánchez et al. (2015), the computational and processing time is a crucial aspect in this kind of work. In this line, the time needed for each step is discussed in the following lines. The alignment process took 68 min and the dense point cloud analysis took 159 min, without taking into account the quiet time needed to upload images and carry out the image georeferencing process. On the other hand, the time required for these steps mainly depends on the covered area, the number of images and their resolution, and the computer used, as suggested by Ai et al. (2015).

Most of the processing time was spent training the Faster R-CNN model, which took approximately 5 h using Google Colab. This depends on the number of images used, the batch size, the learning rate, and the hardware used, among other factors. This step did not take the time required for image labeling, which is highly time-consuming, into account. It usually takes several working days, since it is a process that is completely manual. In a study by Tian et al. (2019a), a similar number of images was obtained using an analogous training time to this study. However, the studies cannot be completely compared, since the hardware and images used were not the same. The research carried out by Id et al. (2019) to detect banana trees on images taken by an UAV took 180 min to train a similar model. They used over 2,000 images with a resolution of 600 × 600 px. However, the CNN architecture used was Yolo-V3, which is slower than Faster R-CNN, according to Rosebrock (2018).

### Assessment of Apple Fruit Detection on UAV Images and Orthomosaics Construction

The main challenge in fruit detection when applying images taken from a UAV is fruit size. In addition, the size of TIF files increases the amount of computational resources needed to train the models. Senthilnath et al. (2016) demonstrated a novel method for detecting tomatoes using UAV images taken with a multispectral camera. They used spectral clustering based on the K-means algorithm to detect tomato fruits. The main problem that they found was the inability to detect fruits covered by the leaves. Other studies, such as that proposed by Id et al. (2019) to detect banana trees, obtained similar results in terms of accuracy to what this study concluded. Hence, we can conclude that the method proposed in this study was highly accurate for fruit detection tasks. In addition, the maps generated from the detections in images taken from a UAV represent an innovative proposal that, until now, has not been implemented in an apple crops field.

Regarding the creation of orthomosaics, many of the tools that make use of them apply segmentation techniques to detect objects (fruits, trees, rows, etc.). Csillik et al. (2018) developed an algorithm for citrus tree identification. They applied the CNN workflow using Trimble's eCognition Developer 9.3 (www.ecognition.com). Johansen et al. (2018) also proposed a methodology using multispectral images to detect tree canopies with the intention of determining the number of trees. Although these methods have a high level of accuracy, the process is not completely automated; hence, it can be improved. On the other hand, much of the research that currently applies DL algorithms operates with individual images without georeferencing (Kamilaris et al., 2017). Knowing the accurate position of each element (plants, machinery, sensors, etc.) available on any farm is crucial (Ramin Shamshiri et al., 2018). To our knowledge, our methodology is the first that allows the orchard yield to be estimated based on the number of fruits detected a tree-scale precision on images taken by an UAV.

**Figure 13:**
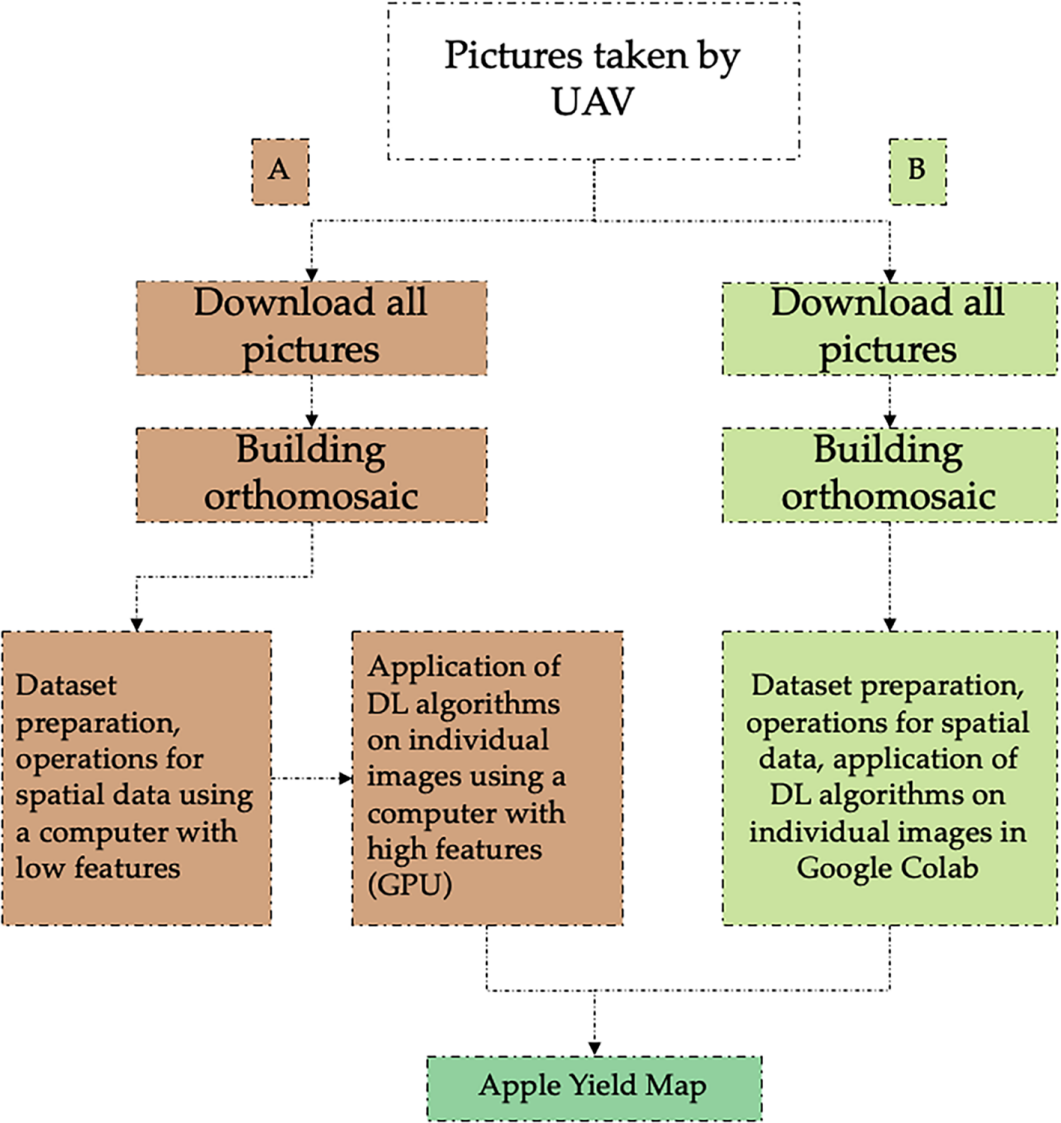
Schematic workflow of the main steps used for orchard yield mapping: **(A)** traditional methodology and **(B)** proposed methodology.

**Figure 13** compares two schematic workflows for the purposes of applying the DL algorithm and other common indexes used in agriculture. On the left (**Figure 13A**), the traditional workflow as used in reference A is presented. This is characterized by the performance of the detection processes on different platforms and in separate steps. For example, the preparation of the datasets is usually done on a conventional computer, while the training of the algorithms is done on a more powerful computer (mainly with advanced GPU hardware). The main advantage of the proposed method (**Figure 13B**) is that Colab allows the data to be prepared and applies fruit detection in georeferenced images on the same platform, which reduces the processing time and leverages the interoperability.

### Integration of Automated Yield Estimation Systems Into the Agricultural Domain

Finally, we would like to focus on the translation of this type of fruit detection and counting systems to the agri-economic terrain. Being aware of the advance that this type of technique implies for an early forecast of yield, we think that it may have an impact on the way in which the management of farms is carried out in the coming years. The organization of harvesting tasks, the pruning of trees, or the fruit purchase process itself can be optimized with this type of system. However, we would like to point out that this type of development, although employing collaborative platforms such as the one shown here, *a priori* does not have the average producer as an end user. We envisage that the development of an automated fruit detection system and the possibility of generating variable crop maps, can be marketed as a service within agricultural cooperatives. When demonstrated in a real environment, with a model with several learning campaigns, it can represent an important advance in the adoption of new agricultural management systems. Although the development in this work implements an open data model, with open-source algorithms, the algorithm-as-a-service model is still far from a firm implementation in the agricultural field. Cloud computing and development platforms such as Google Colab have great potential in the near future to serve as tools for the creation of advanced services in precision agriculture. Moreover, thanks to advances in GPS position enabling farmers to accurately navigate to specific location in the field, a door opens to an automatic harvested in combination with yield maps and autonomous farm equipment's. The object of these developments can be anticipated as they will be integrated into software solutions and much more automated platforms (Farm Management Information Systems), in which the user will hardly have to interact with the data to obtain reliable forecasts.

## Conclusions

This paper introduces a novel methodology for sampling an apple orchard at the tree level to infer the final yield. It was found that it is possible to detect the number of fruits in apple trees from images taken from a UAV. The assessment of the DL model showed very promising values and, therefore, a great potential of the method is foreseen for the estimation of apple yields and probably the yield of other fruits.

Google Colab's usefulness as a tool for training DL algorithms to build useful tools for farmers was assessed. This cloud environment will make the tool more available for further research and improve orchard management. Moreover, the use of python opens the door to developing web tools with the aim of automating the process. In this case we provide the code used in the **Supplementary Material**.

Future works will involve the automation of all of the processes: the creation of the orthomosaic, individual tree identification, the detection of all the fruits in each tree, and the generation of the yield map on a single platform integrated in a graphical user interface (GUI). This will provide stakeholders a useful and easy-to-use tool. Moreover, the combination of historical data from several seasons will be tested to build models where data and images converge to obtain accurate results.

## Data Availability Statement

The datasets generated for this study are available on request to the corresponding author.

## Author Contributions

All authors contributed to the article and approved the submitted version. OEA-A wrote the first draft of the paper and analyzed data. JV conceived the experiments, flew the UAV to take the pictures, and conducted the field measurements. MP-R provided guidance for the analysis of data and writing of the manuscript. JM-G provided suggestions on the structure of the manuscript and participated in discussions of the results.

## Funding

This work was partially supported by the project MARS4Earth: Modular Aerial Robotic Systems for Sustainable Living on Earth (RAAK.PRO03.112), which is funded by the Netherlands Organisation for Scientific Research and the project AGL2016-78964-R funded by the Spanish Ministry of Economic and Competence.

## Conflict of Interest****

The authors declare that the research was conducted in the absence of any commercial or financial relationships that could be construed as a potential conflict of interest.
