# Supplementary material for: A Cloud-Based Environment for Generating Yield Estimation Maps From Apple Orchards Using UAV Imagery and a Deep Learning Technique
Source: Front Plant Sci. 2020 Jul 15;11:1086. doi: 10.3389/fpls.2020.01086 (PMC7378326; doi:10.3389/fpls.2020.01086)
Supplement: Supplementary file 1 [file Table_1.docx]

Supplementary Material

# Supplementary Data

In order to use the code provided in this paper, it is necessary to make a series of previous steps to build the environment where execute the code.

1.- The first step is creating a Google Account to have access to a Google Drive where store all the files necessaries.

2.- The second step is clone inside the folder “models” available in this link: <https://drive.google.com/open?id=1-1zKsp6IJs1Tx4oo9poDgZgV3071npD8>. Inside this folder are all the files necessaries to detect apple fruits in images.

3.- The third step is to upload the file “detectionapple.ipynb”. The file is available in this link: <https://colab.research.google.com/drive/1kK5KqCaXixQnISuisgdj7eBKZ18oea4s>. The user needs to follow the instructions in the file to obtain results.
