# Supplementary figures and images for: A Cloud-Based Environment for Generating Yield Estimation Maps From Apple Orchards Using UAV Imagery and a Deep Learning Technique
Source: Front Plant Sci. 2020 Jul 15;11:1086. doi: 10.3389/fpls.2020.01086 (PMC7378326; doi:10.3389/fpls.2020.01086)

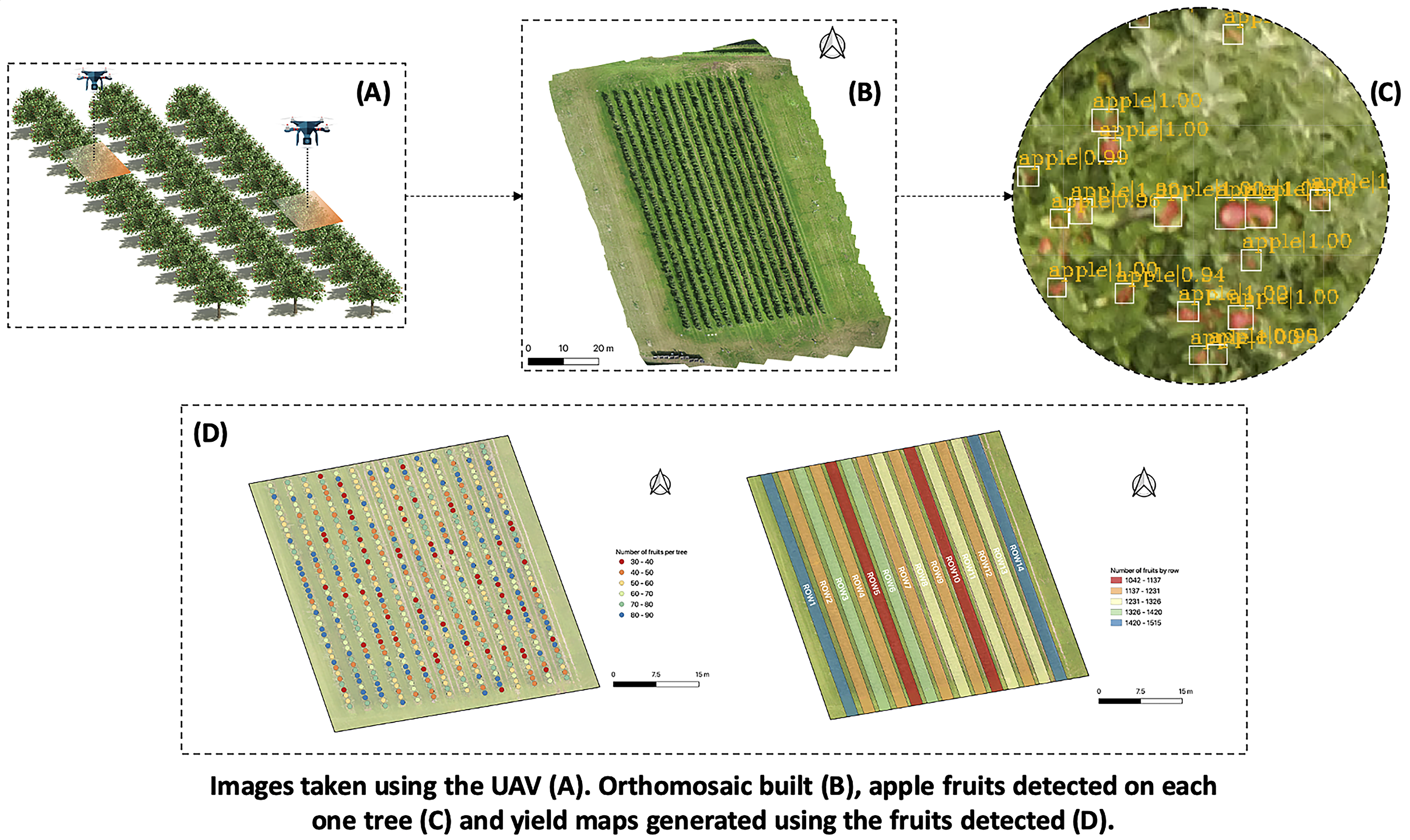

Supplement: Supplementary file 2 [file Image_1.png]
